# Different mechanisms underlie similar species-area relationships in two tropical archipelagoes

**DOI:** 10.1016/j.pld.2023.08.006

**Published:** 2023-09-09

**Authors:** Shengchun Li, Tieyao Tu, Shaopeng Li, Xian Yang, Yong Zheng, Liang-Dong Guo, Dianxiang Zhang, Lin Jiang

**Affiliations:** a Key Laboratory of Plant Resources Conservation and Sustainable Utilization, South China Botanical Garden, Chinese Academy of Sciences, Guangzhou 510650, China; b ECNU-Alberta Joint Lab for Biodiversity Study, Tiantong Forest Ecosystem National Observation and Research Station, School of Ecological and Environmental Sciences, East China Normal University, Shanghai 200241, China; c School of Biological Sciences, Georgia Institute of Technology, Atlanta, GA 30332, USA; d Jiangxi Academy of Forestry, Nanchang 330032, China; e Institute of Eco-Chongming (IEC), Shanghai 202162, China; f State Key Laboratory of Biocontrol, School of Ecology, Sun Yat-sen University, Guangzhou 510275, China; g School of Geographical Sciences, Fujian Normal University, Fuzhou 350007, China; h State Key Laboratory of Mycology, Institute of Microbiology, Chinese Academy of Sciences, Beijing 100101, China

**Keywords:** Coral and continental islands, Plant diversity, Soil nutrients, Species-area relationships, The sampling effect

## Abstract

Despite much research in the field of island biogeography, mechanisms regulating insular diversity remain elusive. Here, we aim to explore mechanisms underlying plant species-area relationships in two tropical archipelagoes in the South China Sea. We found positive plant species-area relationships for both coral and continental archipelagoes. However, our results showed that different mechanisms contributed to similar plant species-area relationships between the two archipelagoes. For coral islands, soil nutrients and spatial distance among communities played major roles in shaping plant community structure and species diversity. By contrast, the direct effect of island area, and to a lesser extent, soil nutrients determined plant species richness on continental islands. Intriguingly, increasing soil nutrients availability (N, P, K) had opposite effects on plant diversity between the two archipelagoes. In summary, the habitat quality effect and dispersal limitation are important for regulating plant diversity on coral islands, whereas the passive sampling effect, and to a lesser extent, the habitat quality effect are important for regulating plant diversity on continental islands. More generally, our findings indicate that island plant species-area relationships are outcomes of the interplay of both niche and neutral processes, but the driving mechanisms behind these relationships depends on the type of islands.

## Highlights

• Explored mechanisms underlying plant species-area relationships in two tropical archipelagoes.
• Increasing soil nutrients availability had opposite effects on plant diversity between coral and continental islands.
• The habitat quality effect and dispersal limitation are important for regulating plant diversity on coral islands.
• The passive sampling and the habitat quality effect are important for regulating plant diversity on continental islands.

## Graphical abstract

**Figure:**
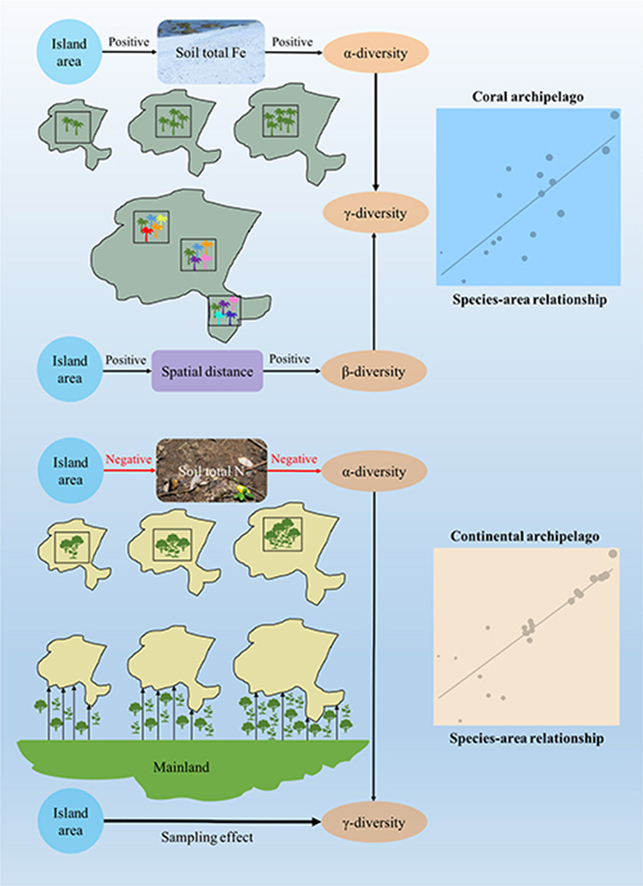

## 1 Introduction

A major challenge in ecology is to understand mechanisms regulating species diversity within and among ecological communities (Ricklefs and Schluter, 1993). Within this conext, islands have long been used as a useful model system for addressing diversity-related questions (Darwin, 1859; MacArthur and Wilson, 1967; Warren et al., 2015; Whittaker et al., 2017). The differences in area, isolation, and environmental characteristics among islands, coupled with their discrete nature, facilitate the investigation of fundamental diversity questions such as why larger areas tend to harbor more species, and what makes assemblages of species different among localities. Nevertheless, despite decades of research, an integrative understanding of community assembly processes that determine species diversity on islands remains largely elusive (Warren et al., 2015; Whittaker et al., 2017).

Three main theories have been proposed to account for species diversity distribution patterns across islands. First, the passive sampling hypothesis suggests that larger islands tend to contain more species, simply because they represent larger targets of species colonization, thus capturing larger species samples from the regional species pool (Connor and McCoy, 1979). Second, the theory of island biogeography (TIB), also known as the area *per se* hypothesis (Connor and McCoy, 1979), emphasizes the balance between species immigration and extinction on islands (MacArthur and Wilson, 1967). TIB proposes that larger and closer islands tend to have higher immigration rates than smaller and farther islands, and that larger islands also tend to have lower extinction rates because of their ability to support larger populations, resulting in higher species diversity on larger and closer islands (MacArthur and Wilson, 1967). TIB has long been appreciated in ecology, biogeography and conservation biology (Higgs, 1981; He and Legendre, 1996, 2002; Desmet and Cowling, 2004; Si et al., 2017). Like the passive sampling hypothesis, this theory does not consider the roles of mechanisms other than neutral processes in driving community assembly on islands (MacArthur and Wilson, 1967). Third, the habitat heterogeneity hypothesis suggests that larger islands tend to have higher species diversity than smaller islands because greater habitat diversity or environmental heterogeneity on larger islands supports more different species across localites (i.e., increased β-diversity within islands) (Williams, 1964; Connor and McCoy, 1979; Sfenthourakis and Triantis, 2009). Much research has assessed the roles of these mechanisms for island species-area relationships, demonstrating their varying importance across taxa and island systems (e.g., Nilsson et al., 1988; Ricklefs and Lovette, 1999; Panitsa et al., 2006; Liu et al., 2020).

The focus on the aformentioned mechanisms in explaining species-area relationships, however, ignores the possibility that other mechanisms may also contribute to these relationships (Li et al., 2020). On the one hand, islands that differ in habitat characteristics may not only differ in habitat heterogeneity but also in their average habitat quality (e.g., soil nutrients), with the latter also having the potential to influence insular species diversity (Schrader et al., 2019). A large body of studies have shown that soil properties could affect plant species diversity (Laliberté et al., 2013). For example, soil N, P, K are three nutrients that are most often limiting, and their effects on plant species richness might be disparate in different ecosystems (Huston, 1980; Janssens et al., 1998; Gusewell et al., 2005; Wassen et al., 2005; Clark and Tilman, 2008; Gilbert et al., 2009; Ceulemans et al., 2014; Goossens et al., 2022). In addition, soil metal micronutrients are essential for plant cell growth and photosynthesis (Welch and Shuman, 1995). For example, iron (Fe) is involved in a variety of biological processes, such as photosynthesis, respiration and chlorophyll biosynthesis, and it is required in the greatest amount among the essential micronutrients in plants (Kobayashi and Nishizawa, 2012). Presumably, soil Fe is important for plant survival and maitaining plant diversity, especially in the environment where soil Fe is insufficient. However, many studies only considered the effects of habitat diversity or heterogeneity on species diversity in island systems (Triantis et al., 2005; Sfenthourakis and Triantis, 2009; Yan et al., 2023), but ignored the impacts of habitat quality on plant diversity, such as the content of soil nutrients in localities. On the other hand, dispersal limitation is also an important mechanism to affect community assembly and diversity (Levine and Murrell, 2003; Shen et al., 2009). Though TIB has considered the dispersal limitation, it emphasizes the distance between islands and mainland. Within-island disperal limitation may be expected to be more important for driving the difference in commumity structure across localities (i.e., larger β-diversity within islands) on larger islands, simply because of the greater distances that organisms need to travel across localities on larger islands. Therefore, among-island difference in habitat quality and within-island dispersal limitation should also be considered when examining species-area mechanisms.

Differentiating the aforementioned mechanisms may be faciliated by decomposing the total number of species on an island (γ-diversity) into species richness at localities (α-diversity) and variation in community structure among localities (β-diversity) within the island (Connor and McCoy, 1979; Schoereder et al., 2004; Li et al., 2020). The passive sampling hypothesis would be supported if γ-diversity, but not α-diversity or β-diversity, is greater on larger islands. As stated above, the habitat quality effect is expected to influence γ-diversity by altering α-diversity, and habitat heterogeneity and dispersal limitation are expected to influence γ-diversity by altering β-diversity.

In this study, we applied the above framework to plant communities in two archipelagoes (Paracel and Wanshan) in the South China Sea to explore mechanisms underlying plant species-area relationships. Paracel and Wanshan archipelagoes represent groups of coral and continental islands, respectively, allowing comparative studies of species-area patterns and mechanisms. The Paracel archipelago is characterized by pospho-calcic and coastal saline soil, and Wanshan archipelago has lateritic red soil. Considering the difference in distance to mainland and environmental conditions (e.g., soil properties) between the two archipelagoes, we hypothesized that the effects of soil nutrients on plant species richness might be different and the species-area relationships might be driven by different mechanisms. However, it remains unclear how soil nutrients affect plant species diversity in island systems, especially in coral islands. In particular, we aimed to address the following questions: 1) what are the relationships between species richness (γ-diversity) and island area and isolation in the two archipelagoes? 2) what are the relationships between species richness (α-diversity) and soil properties in the two archipelagoes? 3) which mechanisms drive the species-area relationships in the two archipelagoes?

## 2 Materials and methods

### 2.1 Study area

**Fig. 1:**
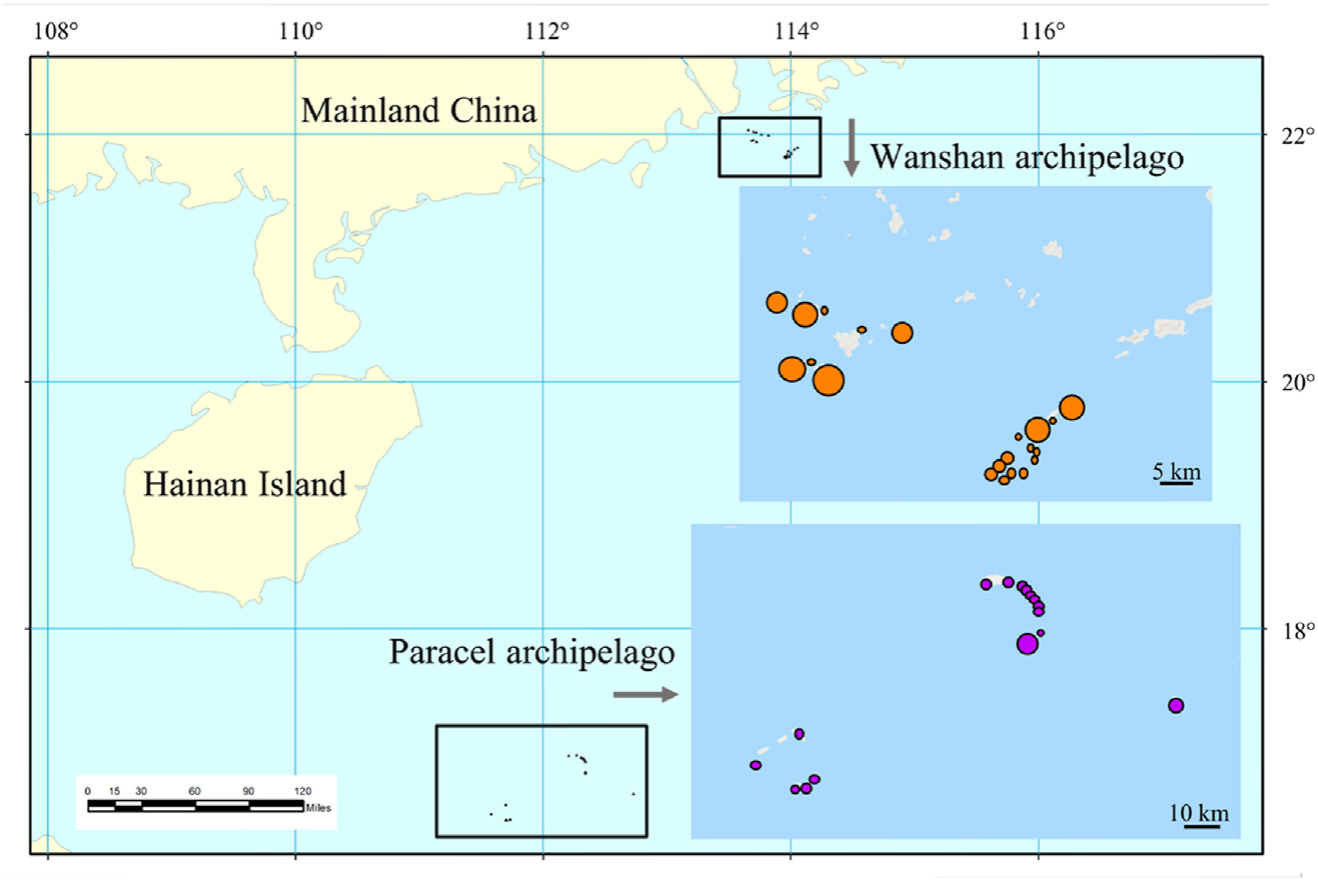
The location of Paracel archipelago and Wanshan archipelago.

**Fig. 2:**
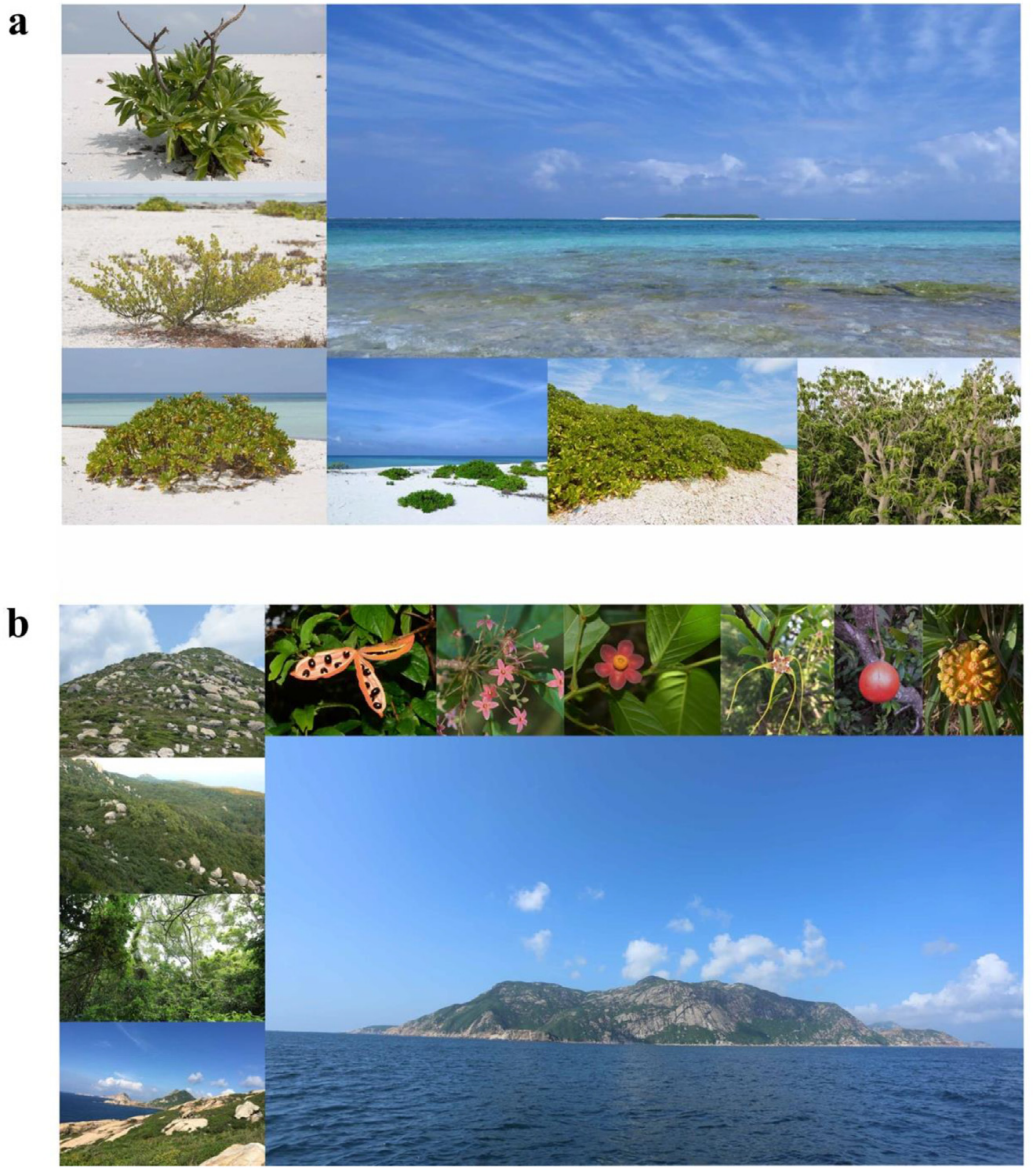
Images of representative islands and their vegetation in Paracel archipelago (a) and Wanshan archipelago (b). Image (a) shows unique soil type and habitat variation on coral islands in Paracel archipelago (Three species on the left: *Tournefortia argentea*, *Suriana maritima* and *Scaevola taccada*). Image (b) exhibits habitat variation on continental islands in Wanshan archipelago, and top of the image (b) shows typical plants in Wanshan archipelago (From left to right: *Sterculia lanceolata-*one of the dominant tree species (Fruit and flower), *Uvaria macrophylla*, *Strophanthus divaricatus*, *Melodinus suaveolens* and *Pandanus tectorius*).

We surveyed 16 coral and 21 continental islands from Paracel and Wanshan archipelagoes, respectively, in the South China Sea from 2014 to 2017 (Fig. 1). The Paracel archipelago (15°46′–17°08′ N, 111°11′–112°54′ E), with an average distance of 330 km from the Hainan Island (the nearest large land mass), is composed of a group of islets ranging from 1 to 210 ha in area and from 1 to 17 m in elevation. The Wanshan archipelago (21°48′–22°11′ N, 113°06′–114°19′ E) has an average distance of 45 km from the mainland, and the islands of Wanshan archipelago range in area from 0.18 to 806.6 ha, and from 8 to 432 m in elevation. The Paracel archipelago is a group of coral islands formed in late Tertiary (Lu et al., 1979; Ye et al., 1985; Gong et al., 1996). The Wanshan archipelago was separated from the mainland in Holocene because of sea level rise (Wang, 2008). The vegetation of Paracel archipelago is mainly composed of shrubs and herbs, with *Scaevola taccada* (Gaertn.) Roxb., *Pisonia grandis* R. Br., and *Guettarda speciosa* L. as the dominant species. By contrast, the vegetation of Wanshan archipelago is characterized by evergreen broad-leaved forests, where *Sterculia lanceolata* Cav.*, Schefflera heptaphylla* (L.) Frodin*, Acacia confusa* Merr.*,* and *Litsea glutinosa* (Lour.) C.B. Rob. are the dominant tree species (Tu et al., 2022). Images of representative islands and their vegetation were provided in Fig. 2.

We surveyed plots of 10 m × 10 m covering different habitats, aspects, and community types for both Paracel and Wanshan islands. More plots were included within each island until the number of recorded species reached a plateau. In the end, we surveyed a total of 589 plots on the 37 islands. All angiosperm species in each of the 589 plots were recorded. Nomencalture followed *Flora Reipublicae Popularis Sinicae* (FRPS) (Editorial Committee of FRPS, 1959–2004) and *Flora of China* (Wu et al., 1994–2013). We recorded 170 and 641 angiosperm species on Paracel and Wanshan archipelagoes, respectively, including one new species (*Quercus pseudosetulosa* Q.S. Li & T.Y. Tu) and one newly recorded genus (*Stillingia* Garden ex Linnaeus) in China (see Li et al., 2017; Li et al., 2018). All the voucher specimens were deposited at the herbarium of the South China Botanical Garden, Chinese Academy of Sciences (IBSC).

### 2.2 Soil properties

We collected soil samples from 195 plots on 18 of the 37 islands; logistic constraints prevented us from sampling all 37 islands. Five soil cores (2.5 cm in diameter, 15 cm in depth) were randomly collected from each plot and homogenized to form a composite sample. Soil total C and total N were measured with an Elementar Vario EL III (Elementar Analysensysteme GmbH, Hanau, Germany). Total Mg, K, Ca, Fe and total P were measured using Inductively Coupled Plasma Optical Emission Spectrometry (ICP-AES, iCAP 6300, Thermo Jarrell Ash Co., USA). Soil pH was measured in soil suspension with a 1: 2.5 (*m/v*) ratio of soil to deionized water, using a benchtop pH meter (Mettler Toledo FE20, Mettler Toledo, Columbus, OH, USA).

### 2.3 Island area and isolation

Data on the area of Paracel and Wanshan islands were obtained from Tong et al. (2013) and Zhuhai Chronicle Committee (1987), respectively. Island isolation was defined as the closest distance to mainland (Wanshan) or to the nearest large land mass (Paracel), measured via Google Earth.

### 2.4 Data analyses

For each island, we defined α-diversity as average species richness within a plot of 100 m^2^, β-diversity as community compositional dissimilarity between plots of 100 m^2^ within the island, quantified by average Jaccard dissimilarity values, and γ-diversity as the total number of species found on the island. Soil heterogeneity within each island was measured as the average pairwise Euclidean distances in the eight soil properties (Soil total C, N, P, K, Mg, Ca, Fe and soil pH) among plots. We also measured spatial distance among plots within island. We used ordinary least squares regression to assess the effect of island area and isolation on γ-diversity for Paracel and Wanshan archipelagoes. We also used ordinary least squares regression to assess the relationships between α-diversity and soil properties, and to measure the effect of island area on soil properties in the two archipelagoes. We then used piecewise structural equation models (SEM) to assess the roles of island area, spatial distance, soil properties and soil heterogeneity for determining α, β and γ-diversity (see Fig. S1). Overall fit of the piecewise SEM was assessed using Shipley's test of d-separation with Fisher's *C* statistic in the *piecewiseSEM* package (Shipley, 2009; Lefcheck, 2016). All statistical analyses were performed in R v.3.5.1 (R Core Team 2018).

## 3 Results

**Fig. 3:**
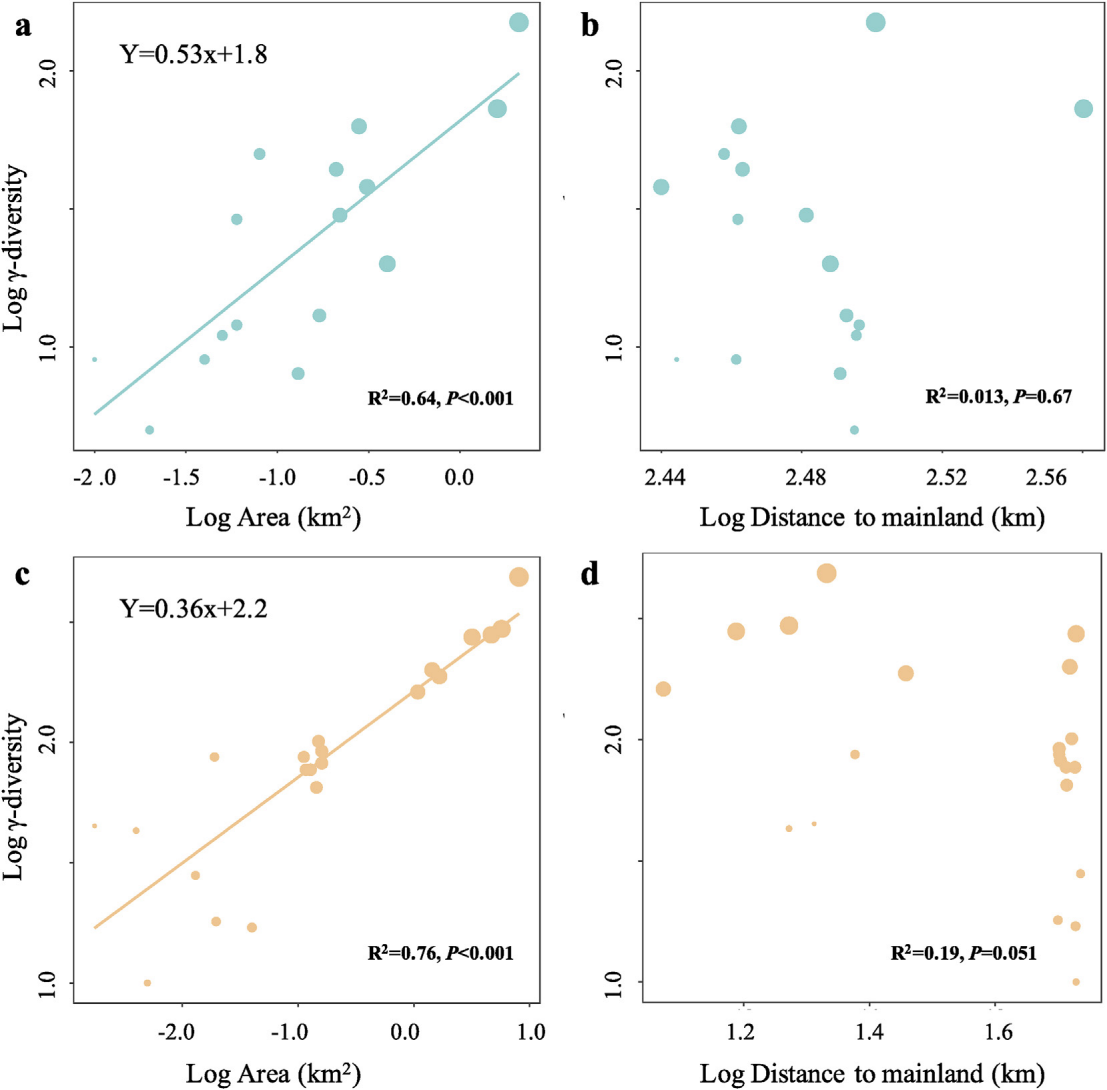
The relationships between plant γ-diversity and island variables (island area and isolation) in Paracel archipelago (a–b) and Wanshan archipelago (c–d).

**Fig. 4:**
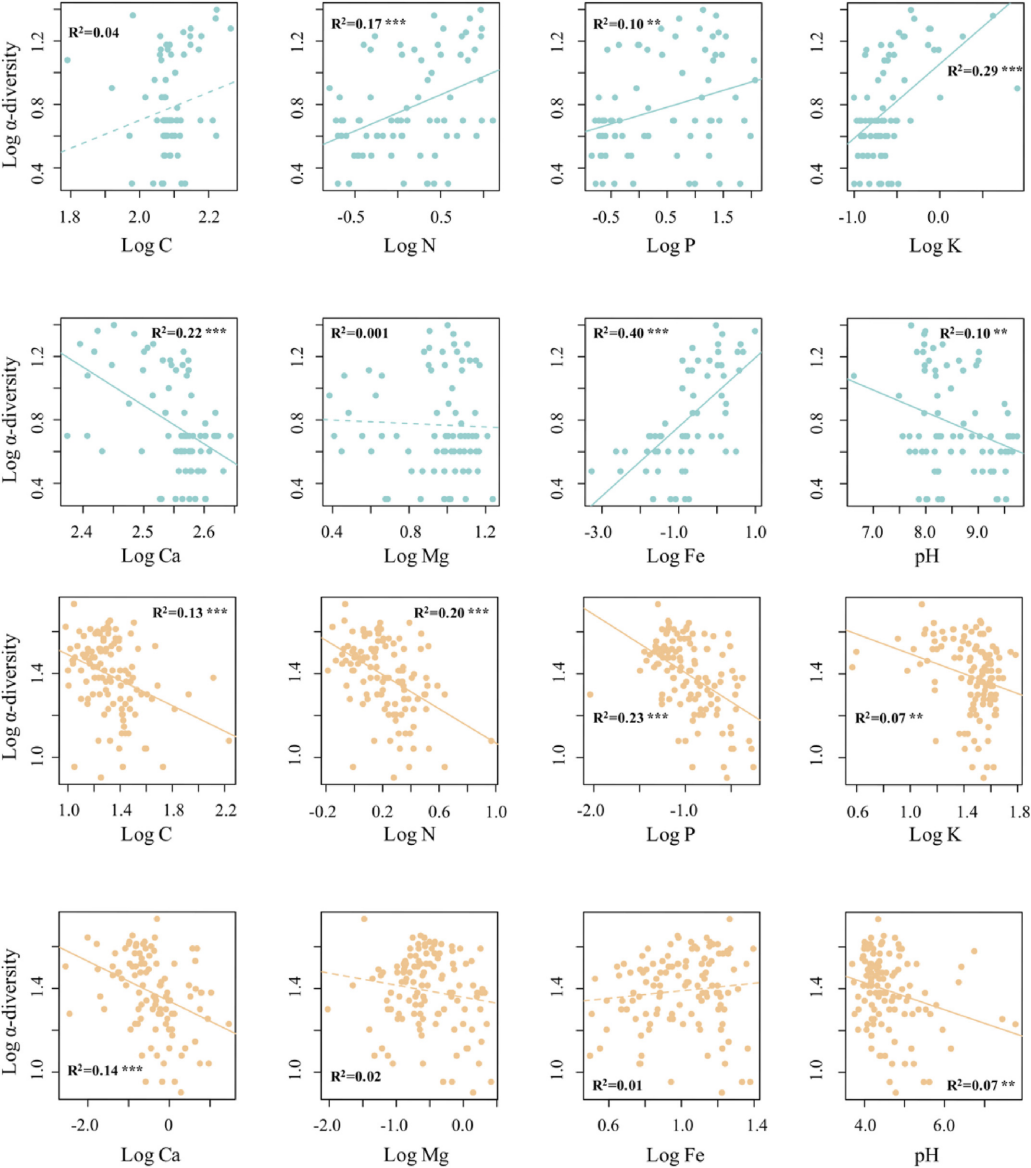
The relationships between α-diversity and soil properties in Paracel archipelago (marked in blue color) and Wanshan archipelago (marked in orange color). *p* < 0.05 ∗, *p* < 0.01∗∗, *p* < 0.001∗∗∗.

We found similar island biogeographic patterns for Paracel and Wanshan archipelagoes (Fig. 3). For both archipelagoes, γ-diversity, measured as the total number of plant species present on an island, showed strong positive relationships with island area (Fig. 3a, c), but no relationships with the distance to mainland (Fig. 3b, d). Intriguingly, we found that the relationships between α-diversity and soil properties showed distinct patterns in the two archipelagoes (Fig. 4). In Paracel archipelago, α-diversity had significantly positive relationships with soil N, P, K, with soil Fe being the best predictor of α-diversity (R^2^ = 0.40∗∗∗). In contrast, soil C, N, P, K showed significantly negative associations with α-diversity in Wanshan archipelago, with soil N and P as the best predictors of α-diversity. Our results also showed that soil Fe, N, K increased with increasing island area in Paracel archipelago, where island area had the greatest effect on soil Fe (R^2^ = 0.79∗∗) (Table 1). In Wanshan archipelago, island area had significantly negative effects on soil N, C and P, with soil N showing the strongest relationship with island area (R^2^ = 0.73∗∗) (Table 1).

**Table 1:** Effect of island area on soil properties in two archipelagoes. Statistically significant results are shown in bold.

Archipelagoes	Soil properties	Estimate	R^2^	*p*-value
Paracel islands	**Fe**	**1.18**	**0.79**	**0.007**
	**N**	**0.68**	**0.53**	**0.03**
	**K**	**0.24**	**0.46**	**0.04**
	pH	−0.80	0.42	0.06
	P	1.04	0.41	0.07
	C	0.02	0.32	0.11
	Ca	−0.03	0.26	0.16
	Mg	−0.07	0.07	0.49
Wanshan islands	**N**	**−0.16**	**0.73**	**0.003**
	**C**	**−0.18**	**0.62**	**0.01**
	**P**	**−0.18**	**0.61**	**0.01**
	Ca	−0.44	0.42	0.06
	K	−0.07	0.30	0.13
	Fe	0.05	0.06	0.54
	pH	−0.12	0.05	0.56
	Mg	−0.09	0.03	0.63

**Fig. 5:**
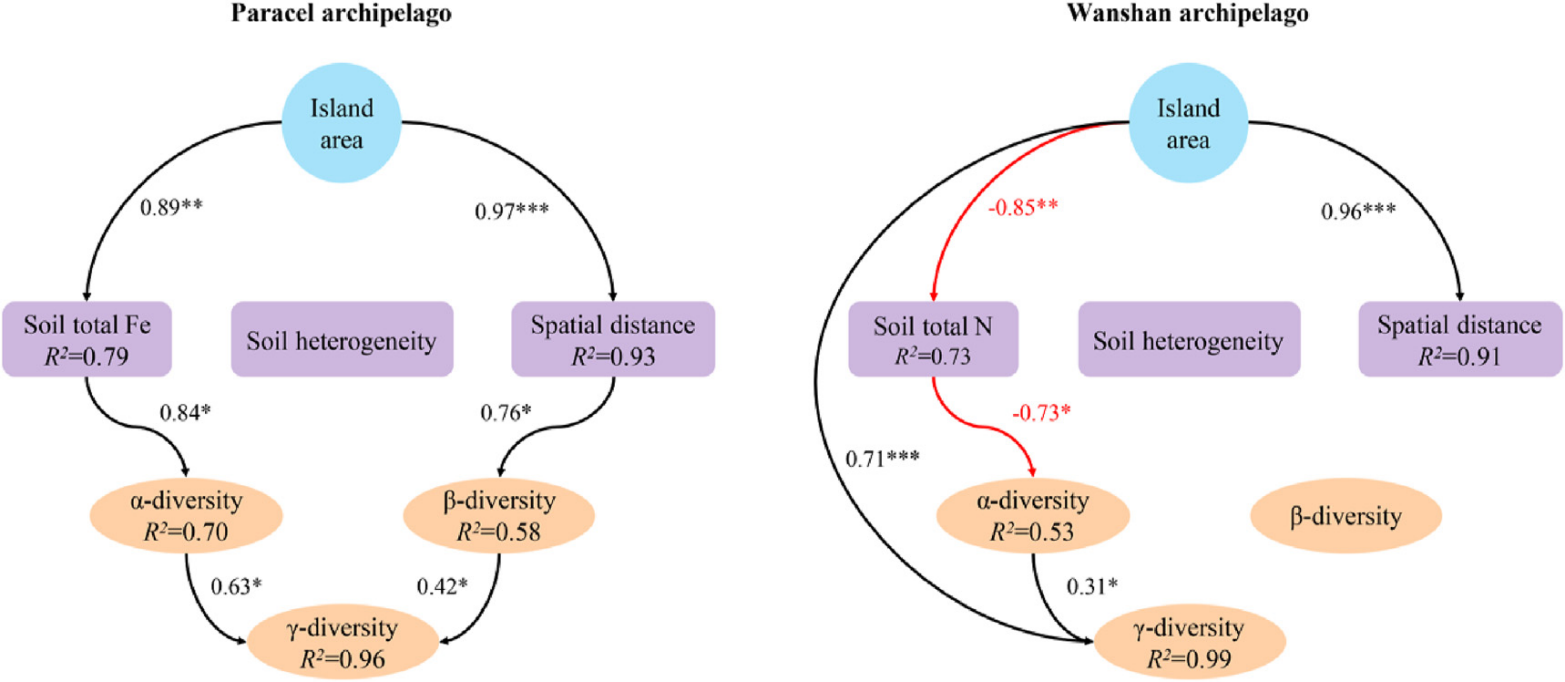
Structural equation models of how island area influences α, β and γ-diversity through different pathways in two groups of archipelagoes. Model of Paracel archipelago (Fisher's C = 32.62; *P* = 0.25), Model of Wanshan archipelago (Fisher's C = 25.17; *P* = 0.12). ∗*P* ≤ 0.05; ∗∗*P* ≤ 0.01; ∗∗∗*P* ≤ 0.001.

SEM identified different drivers of γ-diversity between the two archipelagoes. In Paracel archipelago, both greater α and β-diversity contributed to greater γ-diversity on larger islands (Fig. 5). Greater α and β-diversity on larger Paracel islands were driven by higher soil total Fe and larger spatial distance among plots, respectively (Fig. 5), suggesting the operation of the habitat quality effect and within-island dispersal limitation. By contrast, in Wanshan archipelago, γ-diversity was influenced directly by island area as well as by α-diversity, with the direct effect of island area being much stronger (Fig. 5), indicative of the importance of the passive sampling effect. α-diversity was greater on larger islands with lower soil total N (Fig. 5), pointing to the importance of the habitat quality effect. Unlike Paracel islands, β-diversity did not affect γ-diversity on Wanshan islands (Fig. 5). β-diversity was not influenced by soil heterogeneity on either archipelago (Fig. 5).

## 4 Discussion

TIB predicts that the number of species on islands should increase with island size and decrease with island isolation (MacArthur and Wilson, 1967). However, we found positive plant species-area relationships, but nonsignificant species-isolation relationships, for both Paracel (oceanic) and Wanshan (continental) archipelagoes. Nevertheless, our results are in line with empirical studies reporting more variable and weaker species-isolation relationships than generally positive species-area relationships (Watling and Donnelly, 2006; Giladi et al., 2014; Itescu, 2019; Schrader et al., 2019). A global scale island biogeographic study showed that island species diversity patterns were mainly drived by intra-archipelago processes but not by isolation from mainland (Matthews et al., 2019). Studies on other continental or pacific coral archipelagoes also did not detect significant effect of isolation on plant species richness (Larrue et al., 2018; Liu et al., 2020; Walentowitz et al., 2022). In some studies, nonsignificant species-isolation relationships became significant after accounting for the covariance between island area and isolation (Watling and Donnelly, 2006). This is the case for Wanshan, but not for Paracel archipelago, in our study (see Fig. S2). The close proximity of the islands within Paracel archipelago may have contributed to the lack of distance effects on plant diversity.

Our most important finding is that although the two archipelagoes have similar species-area relationships, the underlying mechanisms are different. We obtained this result by adopting a framework that utilizes island spatial and environmental data to explain species diversity within survey plots (α-diversity) and diversity among plots (β-diversity) within islands. In the oceanic Paracel archipelago, γ-diversity was determined by α- and β-diversity, but not by the direct effect of island area. This result is consistent with a worldwide island biogeographic study, Cabral et al. found γ-diversity is predominantly by indirect abiotic effects on 174 oceanic islands, i.e. acting via the α- and β-components on γ-diversity (Cabral et al., 2014). Both of these two studies revealed the importance of environmental and spatial factors in driving plant species diversity on ocean islands. Here the habitat quality effect arose as larger islands, characterized by higher soil total Fe (Fig. 5), supported higher α-diversity. Our results showed that soil Fe was the best predictor for α-diversity (Fig. 4), and larger islands tended to have higher soil Fe (Table 1). The Paracel archipelago is characterized by pospho-calcic and coastal saline soil with high level of soil Ca (*ca*. 358 g/kg), Mg (*ca*. 9.7 g/kg) and pH (*ca*. 8.5), resulting in harsh and dry soil conditions on islands (Fig. 2a). Fe is slightly soluble under in high pH and calcareous soil conditions (Marschner, 1995). Plants that survived in this extreme enviroment on coral islands had thus undergone strong environmental filtering. Under particularly Fe-poor soil on islands in Paracel archipelago, plant competition for soil Fe on smaller islands might be stronger than larger islands because of lower soil Fe on small islands, resulting in less plant species richness on small islands. Study in Raja Ampat archipelago showed that soil depth emerged as a strong predictor of plant species richness on small islands, pointing to the importance of the habitat quality effect (Schrader et al., 2019). Our study further illustrated the important role of soil nutrients in determining plant diversity on coral islands. Within individual coral islands in Paracel archipelago, the ecological similarity of co-occurring species, conferred by their similar traits as the result of strong environmental filtering, may have promoted the role of dispersal limitation in structuring plant communities across the homogeneously extreme environments. A similar finding has been reported in microbial, within-island spatial distance significantly affected fungal β-diversity but not bacteria (Li et al., 2020). Potentially, differences in community assembly across spatial distance might also be caused by other processes such as competitions with other plants, which would need to be explored by future studies. Together, these results point to the importance of the habitat quality effect and within-island dispersal limitation in regulating the assembly of plant communities, and, in turn, the observed species-area relaionships, in Paracel archipelago.

Unlike Paracel archipelago, the passive sampling effect and, to a lesser extent, the habitat quality effect, drove the species-area relationship in Wanshan archipelago. The Wanshan archipelago is a group of continental islands with an average distance of 45 km from the mainland, making it easier, comparing to the oceanic islands, for plant species to colonize those islands. The importance of the passive sampling effect suggests that larger continental islands in Wanshan archipelago contain more species primarily because they represent large targets for species colonization, and thus sampled more individuals and species from the regional species pool. In addition, Wanshan archipelago used to be parts of the mainland in Late Pleistocene, sea level rise lead to their separation from the mainland in mid Holocene (Wang, 2008). Therefore, larger islands might already have more species than smaller islands when these islands separated from the mainland. The similar predominant role of the passive sampling effect has also been recognized for plant diversity in other island systems (Burns et al., 2010; Schrader et al., 2019). Larger islands have higher species abundances than smaller islands because of more colonists on larger islands, which in turn reduces extinction rates. The significant role of the habitat quality effect in Wanshan archipelago arose as larger islands, which had higher α-diversity, were characterized by lower soil nutrients, particularly soil total N (Fig. 5). Other contiental archipelagoes have also found that habitat quality was important in driving plant species-area relationships (Schrader et al., 2019; Liu et al., 2020). For example, Liu et al. (2020) showed that soil total phosphorus has significantly positive relationship with plant species diversity, suggesting the importance of phosphorus limitation in driving plant diversity. In Wanshan archipelago, however, we found soil nutrients, such as soil C, N, P, K, has significantly negative associations with plant diversity. Wanshan islands are covered by highly weathered, lateritic soils, and larger islands might have lost more nutrients as a result of long-term leaching and erosion, especially for soil N (Table 1). Nevertheless, Wanshan islands may be considered as less selective habitats than Paracel islands, permiting a larger number of plant species to successfully establish their popuplations and broad-leaved forests–which represent late successional stages in this region–to develop on the islands. The diverse plant forms associated with the large species pool, combined with substantial differences in the soil environment among and within the islands, presumably lent to the importance of soil properties in regulating plant α-diversity on Wanshan islands (Fig. 4). Here, the habitat quality effect manifests as plant α-diversity increases on larger islands where soils are less fertile. This result is consistent with the negative relationships between soil fertility and plant species diversity reported for tropical rainforests (e.g., Huston, 1980; Nadeau and Sullivan, 2015), as well as the negative effect of nutrient enrichment on plant diversity in terrestrial ecosystems (Stevens et al., 2004; Clark et al., 2007; Bobbink et al., 2010). A host of hypotheses have been put forward to explain the frequently observed negative soil fertility-diversity relationships (Rajaniemi, 2002), and the exploration of its underlying mechanisms remains an active area of research (Suding et al., 2005; Harpole and Tilman, 2007; Hautier et al., 2009; DeMalach et al., 2017). Moreover, we found γ-diversity is not influenced by soil heterogeneity on both Paracel and Wanshan archipelagoes. This result is consistent with many findings in other island systems. For example, Liu et al. (2018) showed that β-diversity is influenced by neither geographical distance nor island area, and Nilsson et al. (1988) found habitat heterogeneity were uncorrelated to island area. Thus, habitat heterogeneity did not play a role in driving species-area relationships in these archipelagoes.

Furthermore, we found Paracel archipelago had steeper slope for plant species-area relationship than continental archipelago (Fig. 3. Paracel: z = 0.53, Wanshan: z = 0.36), suggesting plant species richness increases with area more quickly in Paracel archipelago than Wanshan archipelago. Paracel archipelago had larger slope than most other remote coral archipelagoes, such as Marshall Islands (i.e. 0.41), French Polynesia and Pitcairn Islands (i.e. 0.31), but less than Cook Islands (i.e. 0.86) (Larrue et al., 2018). By constrast, Wanshan archipelago had a very similar slope with many continental archipelagoes, such as Ionian Islands (i.e. 0.37), Getskar Islands (i.e. 0.38) and 54 islands of Denmark (i.e. 0.34–0.43) (Hannus and von Numers, 2008; Valli et al., 2019; Walentowitz et al., 2022). But there were also some archipelagoes showed smaller slope than Wanshan archipelago, such as Aeolian (i.e. 0.24), Adriatic Islands (i.e. 0.27) and North Sporades (i.e. 0.27) (Nikolić et al., 2008; Iliadou et al., 2020; Chiarucci et al., 2021).

There are some limitations in our study. First, we did not collect soil samples from all the 589 plots on the 37 islands due to logistic constraints. While the existing soil samples were generally evenly distributed on the studied islands, we acknowledge that more robust results would be obtained with the full soil data for all plots. Second, we only measured soil chemical factors, and it is likely that other factors, such as soil physical properties (e.g. soil thickness) and soil topography, could also influence plant community structure and diversity. It remains to be seen how including these additional soil characteristics would influence our results.

## 5 Conclusion

In this study, we found that two archipelagoes exhibited similar positive species-area relationships, but that different community assembly mechanisms were behind these similar relationships. The habitat quality effect and dispersal limitation explained plant species-area relationships in Paracel archipelago (coral islands). For Wanshan archipelago (continental islands), the passive sampling effect and the habitat quality effect were more important in driving the distribution of plant species diversity. Note that although the habitat quality effect was important in both archipelagoes, increasing soil nutrient availability (N, P, K) had opposite effects on plant α-diversity between the two archipelagoes. Moreover, β-diversity contributed importantly to the total number of species richness on coral islands but not continental islands, implying that loss of species diversity caused by local extinction is more likely to happen under extreme environmental conditions (such as coral islands in Paracel archipelago) than benign ones. Overall, these results point to different underlying mechanisms of species-area relationships in coral and continental archipelagoes, illustrating the importance of both neutral and niche processes in driving plant community structure and diversity in island systems, which, if general, would have important implications for the conservation of plant diversity in similar ecosystems.

## Declaration of competing interest

The authors declare that they have no known competing financial interests or personal relationships that could have appeared to influence the work reported in this paper.

## Appendix A Supplementary data

Multimedia component 1 Fig. S1. The priori model of how island area influences α, β and γ-diversity through different pathways. Multimedia component 1

Multimedia component 2 Fig. S2. Correlation between island isolation and residuals which were from a regression of γ-diversity on island in Paracel archipelago (a) and Wanshan archipelago (b). Multimedia component 2
