# Supplementary material for: Different mechanisms underlie similar species-area relationships in two tropical archipelagoes
Source: Plant Divers. 2023 Sep 9;46(2):238–46. doi: 10.1016/j.pld.2023.08.006 (PMC11128831; doi:10.1016/j.pld.2023.08.006)
Supplement: Multimedia component 3 [file mmc3.docx]

**Legend of supplementary materials**

Fig. S1. The priori model of how island area influences α , β and γ-diversity through different pathways.

Fig. S2. Correlation between island isolation and residuals which were from a regression of γ-diversity on island in Paracel archipelago (a) and Wanshan archipelago (b).
